# Corticosteroid Injections for Frozen Shoulder: A Global Online Survey of Health Professionals' Current Practice and Opinion

**DOI:** 10.1002/msc.70078

**Published:** 2025-03-26

**Authors:** Christine Bilsborough Smith, Victoria Ryan, Dave Annison, Melinda Cairns, Rachel Chester, Jeremy Lewis

**Affiliations:** ^1^ Therapy Department Central London Community Healthcare National Health Service Trust London UK; ^2^ School of Health and Social Work University of Hertfordshire Hatfield UK; ^3^ South Tees Hospital NHS Foundation Trust Middlesbrough UK; ^4^ School of Health and Social Sciences Faculty of Medicine and Health University of East Anglia Norwich UK; ^5^ Professor of Musculoskeletal Research Clinical Therapies University of Limerick Limerick Ireland

**Keywords:** corticosteroid injections, frozen shoulder, survey

## Abstract

**Introduction:**

Frozen shoulder is a disabling condition characterised by severe pain and loss of shoulder movement. Corticosteroid injections are targeted at reducing pain in the earlier painful phase. There are multiple studies on the effectiveness of injections for frozen shoulder, but none were identified to assess if this guidance has been translated into clinical practice. The aim of this survey was to investigate the current practice and opinion of musculoskeletal health professionals regarding corticosteroid injections for frozen shoulder.

**Design and Methods:**

The online survey was disseminated via the social media platform ‘X’ (at the time of the survey known as Twitter) over a 5‐week period. Recruitment was by the ‘snowball’ effect. Responses to multiple choice survey questions were analysed with descriptive data. Free text questions were analysed using content analysis.

**Results:**

The number of respondents to the survey was 235. Respondents felt injections have an important role in the management of frozen shoulder (155/235, 66%) and the best time to inject is during the pain predominant phase (191/233; 82%). The glenohumeral joint was the preferred anatomical site to inject (136/235; 58%) with triamcinolone as the preferred steroid (66/155; 43%). A steroid dose of 40 mg/mL was favoured by 55% (83/151) of respondents.

**Conclusion:**

Corticosteroid injections play an important role in the management of frozen shoulder. There was consensus for the type and dose of corticosteroid and anaesthetic; however, the range of preparations used indicated that many decisions may be based on personal preference or local guidelines.

## 1 Introduction

Estimated to impact up to 5% of the population at any point in time (Millar et al. 2022), frozen shoulder (FS) is a debilitating condition characterised by pain and loss of movement (Kelley et al. 2013). Up to 8% of men and 10% of women of working age are thought to be affected (Walker‐Bone et al. 2004). The exact cause of FS remains unclear (Thompson et al. 2022) and in the absence of trauma, specific comorbidity (diabetes, thyroid conditions), or surgery, the term idiopathic or primary frozen shoulder is used. Following spontaneous onset, two overlapping phases of the condition have been described; the pain dominant and the stiffness dominant phases (Lewis 2015). People with diabetes are more likely to develop frozen shoulder influencing recovery and outcome (Dyer et al. 2021).

Management includes wait and watch, non‐surgical and surgical interventions. The aim of treatment is primarily to alleviate pain during daily activities sleeping and to improve function. Surgical interventions include manipulation under anaesthetic and/or arthroscopic capsular release. Non‐surgical treatments, such as physiotherapy and corticosteroid injection, may not be less expensive but are typically quicker to access and have comparable outcomes (Rangan et al. 2020). This research compared one glenohumeral joint injection to the two surgical procedures. Different findings may have been reported if multisite injections were compared due to enhanced outcomes associated with the practice.

The short‐term effectiveness of corticosteroid injection has long been established for pain relief during the painful phase of FS, with early administration recommended in NICE clinical knowledge summaries and clinical studies, 2022 (Challoumas et al. 2020; Maund et al. 2012; A. Rangan et al. 2016; Zhang et al. 2021). Improved external rotation range, may also be observed during the stiff predominant phase (W.‐H. Wang et al. 2024). This encouraging finding requires further investigations. Isolated glenohumeral joint (GHJ), and combined subacromial (SA) injection are the most frequently reported anatomical targets for FS injection (Cho et al. 2022; Cho et al. 2016; Fan et al. 2022; Sharif et al. 2022). The injectate is either a corticosteroid in isolation or in combination with a local anaesthetic. Forty milligrammes of triamcinolone acetate (kenalog) (TA) or methylprednisolone acetate (depomedrone) (MP) are the most frequently used corticosteroids (Liang et al. 2024), but little consensus exists on the most effective drug choice. The addition of local anaesthetic can be both therapeutic for immediate pain relief and helping to confirm diagnosis and/or location (Shah et al. 2019). Lidocaine 1% and 2% concentrations are most commonly reported with volumes of 2 and 4 mLs most often cited (Shah et al. 2019).

Other injection therapy techniques for FS include hydrodistension, suprascapular nerve block, and more recently, sonographically navigated release (Chen et al. 2019; Hopewell et al. 2022; Lee et al. 2023; Rangan et al. 2020; Salt et al. 2019). Intra‐articular injection of TNF‐α (adalimumab) is currently in the early stages of investigation (Hopewell et al. 2022).

There are studies on the effectiveness of corticosteroid injections for FS (Berner 2024; Liang et al. 2024; Y. Wang and Gong 2020) that advocate methylprednisolone or triamcinolone is equally effective when delivered in a dose of 40 mg/mL. No studies were identified to assess whether this guidance has been translated into clinical practice. Although it is established that injection therapy can improve pain and disability in the early stages of FS, no consensus on the pharmaceutical injectate of choice, volume, dose, combination, or anatomical target exists.

This may generate uncertainty for those injecting as well as those considering an injection because of the impact these uncertainties will have on shared decision‐making.

To better understand the role of injection therapy in the management of FS, a survey investigating current injection practice was conducted. The survey aimed to investigate the current practice and opinion of a range of musculoskeletal health professionals who either inject or refer corticosteroid injections for frozen shoulder in terms of medicines, dose and delivery of the injection. Any significant variations from the best available evidence could be identified and further research suggested where applicable.

## 2 Methods

The survey used a cross‐sectional online social media platform ‘X’ (at the time of the survey known as Twitter). The checklist for reporting of an internet‐based electronic survey (CHERRIES) was applied (Eysenbach 2004).

### 2.1 Participants

The target sample was musculoskeletal clinicians who either performed injections or referred for injections for frozen shoulder. Recruitment was targeted at all musculoskeletal clinicians regardless of their country of residence.

### 2.2 Survey Design

The survey was generated using JISC Online Surveys (formerly Bristol Online Survey—BOS) https://www.onlinesurveys.ac.uk/. A 5‐week recruitment period window was opened in the spring of 2022 using the social media platform X. Participants were required to consent before proceeding and were automatically directed at the end of the survey if they did not consent. Participants were asked to share the survey link online upon completion to improve recruitment through the ‘snowball’ effect. No formal sample size calculation was required for this exploratory design survey. The survey questions emerged from themes within the current literature and were presented and explored within a patient and public engagement session. The final survey questions were developed by the lead author (CB) and co‐author (JL), who each has over 25 years of experience in musculoskeletal conditions. Closed questions were used and where appropriate, open questions were used. There were 38 questions in total. The first set of questions collected data on profession, years qualified, type of clinic setting, and country of residence. The second set of questions focussed on the number of cases of frozen shoulder seen in a defined time period, how the individual clinician viewed the importance of injection therapy, and whether the clinician performed injections or was referred for injection therapy. The third stage explored details of the injectate in terms of dose, the type of steroid/anaesthetic and injection site(s). The survey took approximately 10 min to complete; The questions are available as Supporting Information S1 data on request of the authors.

### 2.3 Ethics

Ethical approval was granted by University of Hertfordshire United Kingdom Research Ethics Committee Ref: HSK PRG UH 04693. The purpose of the study and informed consent was provided at the start of the survey at which point respondents could cease involvement if they chose. Potential respondents were informed that the survey was anonymous as no participant identifiable information was gathered. Detailed information was provided to enable respondents to understand the time commitment for responding and how far they had progressed when responding.

### 2.4 Data Analysis

Survey data were extracted from the survey website into Microsoft Excel (Microsoft Corp, Redmond, WA, USA). Descriptive statistics were used to analyse the data using the Statistical Package for the Social Sciences (SPSS) version 28 (https://www.ibm.com/spss). Data are presented as frequencies and percentages. Free text questions were analysed using content analysis (Kleinheksel et al. 2020). It was not possible to provide a response rate as the survey was on a single online platform and the survey could have been viewed by individuals to whom the survey was not relevant, and to those that it was, but chose not to participate.

### 2.5 Results

Two hundred and thirty‐five (235) respondents, from 33 different countries, across six continents participated in the survey. Table 1 presents a breakdown of the number of survey participants and their country of origin.

**TABLE 1:** Respondents by country.

Country	Total *n* = 235 (%)
United Kingdom	132 (56.2)
Ireland	12 (5.10)
United States of America	11 (4.7)
Australia	11 (4.7)
Italy	8 (3.4)
Netherlands	6 (2.6)
France	5 (2.1)
Spain	5 (2.1)
New Zealand	5 (2.1)
Argentina	3 (1.3)
Belgium	3 (1.3)
Canada	3 (1.3)
India	3 (1.3)
Norway	2 (0.9)
Germany	2 (0.9)
Singapore	2 (0.9)
Indonesia	2 (0.9)
Austria	2 (0.9)
Finland	2 (0.9)
Denmark	1 (0.4)
Israel	1 (0.4)
Taiwan	1 (0.4)
Zimbabwe	1 (0.4)
Chile	1 (0.4)
China	1 (0.4)
Switzerland	1 (0.4)
Panama	1 (0.4)
United Arab Emirates	1 (0.4)
Saudi Arabia	1 (0.4)
Brazil	1 (0.4)
Kuwait	1 (0.4)
South Africa	1 (0.4)
Nigeria	1 (0.4)
No response	2 (0.9)

## 3 Respondents

Nine different professions responded. Physical therapists and physiotherapists represented the largest professional group (*n* = 213/235, 90%) with over half of all respondents residing in the United Kingdom (*n* = 132/235, 56%). Other professional representation included 10 orthopaedic surgeons, four sports and exercise medicine doctors, three osteopaths, two physiatrists, one myotherapist, one radiologist, and one sonographer. The number of years respondents were qualified ranged from five to 25 years, with almost 80% of respondents reporting 10 years of qualification or more. Respondents worked in a variety of healthcare settings with nearly 40% in private practice (*n* = 88/235, 37.4%) and 23% in secondary care (*n* = 55/235). Table 2 presents this information.

**TABLE 2:** Demographics of respondents.

	Total *n* = 235 (%)
Healthcare profession	
Physiotherapist	197 (83.8)
Physical therapist	16 (6.8)
Orthopaedic surgeon	10 (4.3)
Sports and exercise medicine doctor	4 (1.7)
Osteopath	3 (1.3)
Physiatrist	2 (0.9)
Radiologist	1 (0.4)
Sonographer	1 (0.4)
Myotherapist	1 (0.4)
Years qualified	
< 5 years	22 (9.4)
5–10 years	27 (11.5)
11–15 years	63 (26.8)
16–20 years	35 (14.9)
21–25 years	45 (19.1)
> 25 years	43 (18.3)
Workplace setting	
Private practice	88 (37.4)
Secondary care	55 (23.4)
Primary care	50 (21.3)
Community care	31 (13.2)
Other	11 (4.7)

## 4 Clinical Condition Exposure

The majority of respondents see one to two people diagnosed with frozen shoulder a week (154/235; 66%). One individual working within secondary care reported seeing more than 15 people a week, whilst a l proportion of respondents (19/235; 8%) neither assessed nor referred people with frozen shoulder for injection.

## 5 Practice

**CHART 1:**
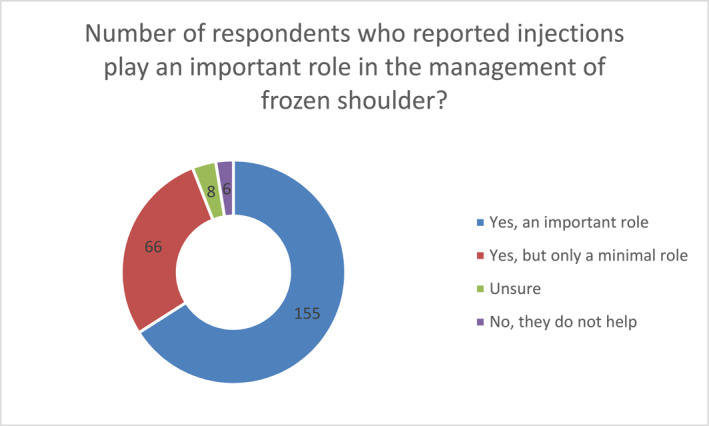
The role injections play in the management of frozen shoulder.

Irrespective of post‐qualification years and place of work, the majority of survey respondents believed injection played an important role in the management of frozen shoulder 66% (155/235) (see Chart 1). A further 28% (66/235) felt injection had a minimal role, whereas 3% (6/235) did not support their use in the management of FS.

A total of 198 respondents (198/235; 84%) either practiced or referred for injection therapy, with just over half referring for the intervention (111/198; 56%).

## 6 Phase of Condition to Inject

Two respondents did not answer this question. Eighty‐two percent (191/233) believed that the pain predominant phase was the most beneficial time to inject, whilst the remainder of respondents felt injections could be performed at either; any phase (33/233; 14%), the stiff predominant phase (2/233; 1%) or were not appropriate at any phase (7/233; 3%).

## 7 Use of Image Guidance

Two hundred and thirty‐one (231) participants responded to this question.

The greatest number of respondents felt injections should be delivered under ultrasound guidance (94/231; 41%) with the second highest response advocating that any method was acceptable (70/231; 30%). Landmark guided was advocated by 18% (41/231) whilst few supported the use of fluoroscopy (8/231; 3%).

## 8 Injection Site(s)

GHJ was the preferred anatomical site to inject (136/235; 58%), with combined GHJ and SA second most preferred (72/235; 31%) and SA alone was the third preferred option (10/235; 4%). One respondent indicated the coracoacromial ligament and coracohumeral ligament (1/235; 0.43%) whilst two reported the suprascapular notch, indicating the use of a suprascapular nerve block (SSNB).

## 9 Glenohumeral Joint Injection

### 9.1 Corticosteroid

Six different steroid preparations were reported. Most respondents used TA (66/155; 43%), followed by MP (38/155; 25%), MP 1% premixed (22/155; 14%), and MP 2% premixed (8/155; 5%). Betamethasone and Adcortyl each had a single response.

A steroid dose (mg/mL) of 40 mg/mL was most frequently reported (83/151, 55%), ranging from 10 mg/mL to 80 mg/mL. A total steroid volume of 1 mL was most frequently reported (87/145; 60%) but ranged from 0.25 to 5 mL.

### 9.2 Anaesthetic

**CHART 2:**
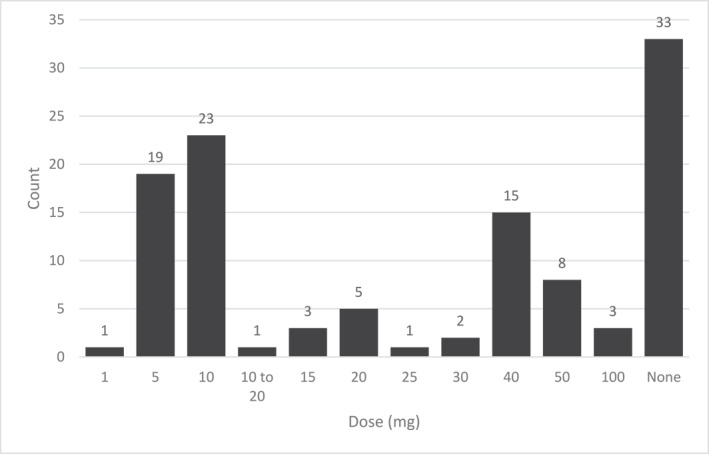
Lidocaine dose (mg) for glenohumeral joint injection.

Most respondents chose lidocaine as their anaesthetic of choice (109/155; 70%), with 10 mg most often selected (23/114; 20%), followed by 5 mg (19/114; 17%) and 40 mg (15/114; 14%). Dose ranged from 1 to 100 mg and volume ranged from 1 mL to 10 mLs. Chart 2 details the range of anaesthetic doses for the glenohumeral joint. A total of 41 respondents chose not to incorporate local anaesthesia in their injection, with one respondent adding: ‘*I use water as opposed to anaesthetic to provide volume to the injection…(to)…minimise potential adverse effects of anaesthetic*’. Bupivacaine was selected by 19 respondents (19/155; 12%), whilst 25 (25/155; 16%) favoured no anaesthetic.

When all anaesthetic preparations were considered, most respondents favoured 5 mLs (33/141; 23%), followed by 4 mLs (26/141; 18%) and then 10 mLs (23/141; 16%) with volumes ranging from 1 mL to 50 mLs.

## 10 Subacromial Space Injection

### 10.1 Corticosteroid

Five different steroid preparations were reported. The most frequently reported was TA (44/133; 33%) followed by MP (30/133; 23%), MP 1% premix (19/133; 14%) and MP 2% premix (6/133; 5%). Two respondents (1.5%) favoured triamcinolone hexacetonide (aristospan). A steroid dose (mg/mL) of 40 mg/mL was most frequently reported (52/132; 40%) ranging from 10 mg/mL to 40 mg/mL. A total steroid volume (mL) of was 1 mL was most frequently reported (55/126; 44%) but ranged from 0.25 to 1 mL.

### 10.2 Anaesthetic

Two anaesthetic preparations were reported: lidocaine (79/127; 62%) and bupivacaine (16/127; 13%). A total dose of 10 mg was reported across 18% (22/119) of participants using local anaesthetic, whilst 5 and 40 mg were reported across 12% and 10% of participants, respectively. A volume of 5 mLs was reported across 23 respondents (23/123; 19%), followed by 4 mL (2/123; 16%) and 10 mLs (12/123; 10%). Dose ranged from 0.25 to 100 mg and volume ranged from 1 mL to 15 mLs. A total of 41 respondents favoured no anaesthetic (41/119; 34%).

## 11 Free Text

The topic most frequently commented upon was in relation to the use of hydrodilitation (HD). These ranged from performing HD instead of a low volume corticosteroid, to considering HD in the stiff phase. Others responded that the volume required varied but must lead to rupture of the capsule. One respondent felt that HD is as effective as manipulation under anaesthesia, whilst another felt HD was a useful adjunct primarily in the stiff phase, whereas another reported that HD had an overall greater role to play than low volume injections in the management of FS.

## 12 Discussion

This paper presents the practice and opinion of 235 healthcare professionals, across nine professional groups, from 33 countries who responded to an online survey on the X social media platform on injection therapy for frozen shoulder. Most respondents felt injection therapy was of value within the pain predominant phase of FS, and those who did inject had a propensity to do so at the glenohumeral joint, typically with a combination of TA and lidocaine.

The research literature supports the early administration of corticosteroids during the painful phase of FS (Ahn et al. 2018; Favejee et al. 2011; Kraal et al. 2018), a position mirrored by the respondents to this survey. Despite recent cellular level studies (Ng et al. 2024), there remains a lack of understanding of both the pathophysiology of FS and its treatment. Although not certain, research has suggested that corticosteroids may alter the course of FS due to the reduction in fibromatosis, vascular hyperplasia, fibrosis, and fibroblasts present in FS in the early stages (Hettrich et al. 2016). Interestingly, 33 respondents (33/233; 14%) felt injections could be performed at either phase, whilst two felt benefit also within the stiff predominant phase (2/233; 1%). A recent retrospective cohort study of ‘frozen‐phase’ participants, those with limited passive ROM (<50% compared to the uninvolved side) of four months or more, has reported statistically significant therapeutic effects of two consecutive GHJ corticosteroid injections, six weeks apart, during the stiff phase (W.‐H. Wang et al. 2024).

Despite a lack of strong evidence of improved outcomes with Ultrasound Guided injection (Zadro et al. 2021), 40% of respondents favoured its use, whilst another 30% advised any method was acceptable. Interestingly, one respondent advised a landmark injection for a single low volume injection whilst another, presumably to avoid delay in access to treatment, advised landmark guided in the painful stage, but ultrasound guided in the latter stages, although no further detail was provided.

One comment ‘depends on clinician skill’ and another ‘landmark by shoulder surgeon or ultrasound otherwise’ highlight potentially both the need for greater competence but also relevance of needle placement accuracy. Two recent studies, one in which fellowship trained orthopaedic surgeons delivered the injection, demonstrated high needle placement accuracy of up to 94% with anterior approach landmark guided intra‐articular GHJ injections (Bartels et al. 2024; Rijs et al. 2021). Despite such findings, a 2022 survey highlighted that both patients and clinicians alike reported greater confidence in the injection procedure if it was conducted under ultrasound guidance (de la Torre‐Aboki et al. 2022).

Nearly two thirds of respondents favoured an isolated injection to the GHJ, whereas one third preferred a combined GHJ and SA injection. A study demonstrated that combined injection at the GHJ and SA space marginally improved internal rotation greater than either location alone (Cho et al. 2016). Significant improvements in short term pain and function, including internal rotation in the longer term, were also reported from multisite injections (Fan et al. 2022; Shang et al. 2019). Conversely, GHJ injections alone have demonstrated significant improvements in pain in the longer term (Chen et al. 2019). It should be noted that with study variation in drug choice, dose and volume across studies, it is difficult to draw direct and definitive comparisons. High quality evidence is yet to demonstrate meaningful difference between anatomical choice of injectate delivery, and whether single site or combined is of greater value, but respondents in this survey favoured a single site injection over a combined approach. A single site injection may be more practicable in a clinical setting unless more complex injections, such as the coracoacromial and coracohumeral ligaments and suprascapular nerve, are sought.

In keeping with similar studies (Livadas et al. 2024; Salt et al. 2019), most respondents (*n* = 66) favoured the use of TA for GHJ injection. A recent network meta‐analysis study found both TA and MP to be equally effective and supported the use of either (Liang et al. 2024). Although the specific mechanism by which glucocorticoids act upon tissue is not fully understood (Hardy et al. 2020), the properties of TA mean it is less soluble than other common corticosteroids, remains at site longer, and potentially has a lengthened therapeutic window of up to 21 days (Shah et al. 2019). A systematic review and meta‐analysis of five studies comparing 1 mg/mL 40 mg TA to four studies of 1 mg/mL 40 MP in the treatment of FS (Shang et al. 2019) showed no difference for pain or function, a finding also supported by a randomised controlled trial (Carroll et al. 2018). Just over one third of respondents also favoured TA for SA space injection, with 1 mL of 40 mg/mL reported by 44% and 40% of respondents.

Survey respondents also chose to inject MP, and in all its forms, was marginally the overall steroid of choice for GHJ (*n* = 68) and SA space (*n* = 55). Thirty of those choosing MP for GHJ and 25 for SA space chose to utilise a premix of MP and lidocaine. No free text data was collected on this response but could be reflective of both ease in terms of safety and standardisation as no mixing of drugs is required, and the changing of syringe of the different medications is also not required. Non‐prescribing clinicians within the United Kingdom (UK) are not permitted to mix medicines within the same syringe as this is said to create a new ‘off licence’ drug according to Medicines and Healthcare products Agency, which is the UK's national body that regulates the safety and quality of medicines, medical devices and blood components. Interestingly, a UK survey of physiotherapy injection practice highlighted that availability in clinic was the most common reason for corticosteroid choice (Livadas et al. 2024). No high quality sufficiently powered randomised controlled trial compares TA and MP in the treatment of FS and further work is required. Clinician choice within this survey may reflect the multifactorial factors around medicine choice of ease and access.

Lidocaine was the preferred local anaesthetic of choice for both GHJ and SA space injection with 109 and 79 responses, respectively. Lidocaine is both a fast and short acting cost‐effective anaesthetic with a relatively low risk profile compared to other anaesthetics (Balakrishnan et al. 2015). Local anaesthesia injection can be both diagnostic and therapeutic. For the injectate to be diagnostic, it must be administered to the target anatomical site and pain is reduced; it potentially provides clinicians with information on injection location accuracy, although this is disputed due to the dispersion of the injectate (Wu et al. 2022). Local anaesthetic may also be chosen to add volume to an injection and may help reduce pain (Cook et al. 2018). Despite lidocaine as the anaesthetic of choice, when pooled analysis is undertaken, no additional benefit for pain and range of movement is noted beyond 2–3 weeks (Shang et al. 2019).

Across survey respondents, 33 and 41 individuals specifically chose not to use local anaesthetic for GHJ and SA injections which is similar to those found in a recent survey (Livadas et al. 2024). One respondent highlighted ‘potential adverse effects of anaesthetic’ as their reason for exclusion. Serious immediate adverse effects can include anaphylaxis as well as local anaesthetic system toxicity, although with the doses and volumes reported within the survey this would be unlikely. Healthcare professionals working within a community setting with minimal medical support and equipment may choose to avoid the addition of local anaesthetic to reduce the risk of a serious adverse event. Evidence of potential delayed deleterious effects with repeated corticosteroid injections has also increased. Lidocaine and bupivacaine have been reported to both reduce tenocyte numbers and collagen organisation, subsequently impacting tendon physiology (Cook et al. 2018). Repeated steroid injections have been reported to accelerate osteoarthritis progression, osteonecrosis and joint destruction (Kompel et al. 2019), potentially accelerating the need for arthroplasty (Burnett et al. 2021). As no data is available on respondents' choice to omit local anaesthetic, the acceptability of risk around injection therapy may be a topic worthy of further investigation.

Whilst the evidence does not support or refute the use of HD in frozen shoulder (Cook et al. 2022), respondents claimed to have anecdotally good outcomes and there was a call for further research in this area. As seen within this survey, little consensus exists in the ‘optimum’ injectate for use in those with FS. A modified Delphi consensus on the use of HD in idiopathic FS published in 2022 highlighted that the use of image guidance, (isotonic) saline, local anaesthetic and steroids was encouraged, whilst hypertonic saline was ‘disallowed’ (Thompson et al. 2022). A recently published retrospective audit on the use of HD for FS demonstrated reduced onward referral to orthopaedics following the introduction of an HD pathway for FS (Whelan et al. 2023). Conversely, 33% who underwent HD in the stiff predominant phase required further treatment for their FS (Flintoft‐Burt et al. 2023).

Two respondents also highlighted the use of suprascapular nerve blocks (SSNB) in the management of FS. Although the results of a recent RCT comparing GHJ versus SSNB in FS are yet to be published (Jump et al. 2023) a recent randomised double‐blind placebo‐controlled trial of SSNB intervention and placebo demonstrated that SSNB reduced the duration of FS and resulted in reduced pain and disability experienced (Shanahan et al. 2022). An observational study of combined SSNB and HD has also demonstrated improved pain and function scores for FS (Albana et al. 2022). A recent large retrospective study supports such findings and concludes that ultrasound guided combined SSNB and HD supplemented with physical therapy is a safe and effective treatment in reducing pain and improving range of movement in those with FS (Albana et al. 2022). Conversely, a small recent randomised trial concluded that SSNB alone was an effective treatment in improving abduction and external rotation range, whilst the addition of HD did not yield additional benefit (Mulkoglu et al. 2024).

## 13 Strengths and Limitations

Participants from 33 countries across six continents contributed to the results of this survey. A key strength of this survey is that it represents international opinion and practice of injection therapy for FS, presenting and acknowledging the diversity of global practice. Although a larger percentage of responses were submitted from the UK, this is reflective of similar studies on injections for shoulder pain (Salt et al. 2019) and may also reflect that injection therapy plays a significant role in UK physiotherapy management for FS, which made up a significant proportion of overall respondents. Although this majority may lead to bias in one profession, according to the Chartered Society of Physiotherapy, injection therapy has been within the scope of practice for UK Physiotherapists since 1997, and their opinion is likely to reflect UK practice (Chartered Society of Physiotherapy 2023).

A unique aspect of this survey is that it captures the practice and opinion of healthcare professionals who do not undertake injections but who do so. As it is not always necessary to be able to perform a procedure to be aware of and understand the evidence around said procedure, we have included their data to share their knowledge and insights. This may have contributed to the large number of respondents who participated in this survey. Although no formal sample size was calculated, the number of respondents is comparable to other large surveys (Dennis et al. 2017; Hanchard et al. 2011). It is however possible in this study that some of the responders were not qualified and registered healthcare professionals as profession was self‐reported. A review of the data suggested this may involve a single respondent and we could be fairly certain this would not have affected outcomes. Thirdly, some respondents referred to volume in the dose section and vice versa and may reflect uncertainty or understanding of the terms. Finally, this survey was conducted in early 2022 during the Covid‐19 pandemic and may not reflect pre‐ or post‐pandemic practice.

## 14 Conclusion

Most respondents felt injection therapy was of value within the pain predominant phase of FS, and those who did inject most commonly did so with a combination of TA and lidocaine at the glenohumeral joint. Uncertainty in the literature surrounding injectate, location, use of imaging and stage of FS appears to be reflected in the survey responses and highlights the need for consensus and/evidence—based standardisation of injection practice for FS. Fewer respondents reported the use of hydrodilatation and suprascapular nerve blocks in FS but this practice should be included in future studies given its growing clinical use.

## Author Contributions

**Christine Bilsborough Smith:** conceptualisation, methods, formal analysis, data curation, writing – original draft, writing – review and editing. **Victoria Ryan:** formal analysis, data curation. **Jeremy Lewis:** conceptualisation, writing – review and editing. **Dave Annison:** writing – review and editing. **Melinda Cairns:** writing – review and editing. **Rachel Chester:** writing – review and editing.

## Conflicts of Interest

David Annison is a tutor on a masters accredited injection therapy course at Teesside University and for ultrasound guided injections for Sports Medicine Ultrasound Group (SMUG). No other authors have competing interests.

## Supporting information

Supporting Information S1

## Data Availability

The data that support the findings of this study are available from the corresponding author upon reasonable request.
