# Supplementary material for: Corticosteroid Injections for Frozen Shoulder: A Global Online Survey of Health Professionals' Current Practice and Opinion
Source: Musculoskeletal Care. 2025 Mar 26;23(1):e70078. doi: 10.1002/msc.70078 (PMC11946008; doi:10.1002/msc.70078)
Supplement: Supplementary file 1 — Supporting Information S1 [file MSC-23-e70078-s001.docx]

**Survey title: Corticosteroid Injections for frozen shoulder: an online survey of health professionals’ current practice and opinion**

**The survey took approximately 10 minutes to complete**

**Questions**

1. What is your healthcare profession?

| Chiropractor |
| --- |
| General practitioner |
| Nurse |
| Orthopaedic surgeon |
| Osteopath |
| Pain specialist |
| Physiatrist |
| Physiotherapist |
| Physical therapist |
| Radiologist |
| Rheumatologist |
| Sports and exercise medicine specialist |
| Other |

1. How long have you been practicing in your current profession?

| Less than 5 years |
| --- |
| 5 to 10 years |
| 11 to 15 years |
| 16 to 20 years |
| 21 to 25 years |
| More than 25 years |

1. Where is your main place of work?

| Private practice |
| --- |
| Primary care |
| Community care |
| Secondary care |
| Other |

1. In which country do you currently practice healthcare?
2. On average how many people diagnosed with frozen shoulder do you see each week? (select the most appropriate)

| None |
| --- |
| 1 to 2 |
| 3 to 4 |
| 5 to 10 |
| 11 to 15 |
| More than 15 |

1. Whether you inject or not, do you think injections have a role in the management of frozen shoulder?

| Yes, an important role |
| --- |
| Yes, but only a minimal role |
| No, they do not help |
| Unsure |

1. Please tick the most appropriate response

| I perform injections |
| --- |
| I sometimes inject and I also refer people with frozen shoulders for injections |
| I don’t inject but refer people with frozen shoulders for injections |
| I don’t inject and I don’t refer people with frozen shoulders for injections |
| Other |

1. When is the best time to offer an injection?

| Pain greater than stiff phase |
| --- |
| At any phase |
| Not appropriate at any phase |
| Stiff greater than pain phase |

|  |
| --- |
| 1. Whether you inject or not, how should frozen shoulder injections be performed?  \| Landmark guided \| \| --- \| \| Ultrasound guided \| \| Fluoroscopy guided \| \| Injections are never appropriate and should not be performed \| \| Any method is acceptable \| \| other \| |
|  |
| 1. Whether you inject or not, in your opinion which structure(s) should be injected?  \| The glenohumeral joint \| \| --- \| \| The subacromial space \| \| The glenohumeral joint and subacromial space \| |

1. Whether you inject, or not, we are interested in your opinion regarding what medicines (if any) should be used in an injection for the glenohumeral joint.

| I have no opinion |
| --- |
| Glenohumeral joint injections are never appropriate and should not be given  I would inject, recommend or refer a patient with frozen shoulder for injection |

1. Corticosteroid?

| If you inject the glenohumeral joint please type; medicine, dose, volume |
| --- |
|  |

| If you inject the subacromial bursa please type; medicine, dose, volume |
| --- |
|  |
| Other: please type in any other medicines, supplements, procedures you perform |
|  |

1. Anaesthetic?

| If you inject the glenohumeral joint please type; medicine, dose, volume |
| --- |
|  |

| If you inject the subacromial bursa please type; medicine, dose, volume |
| --- |
|  |
| Other: please type in any other medicines, supplements, procedures you perform |
|  |

1. Free text

If you have any other comments regarding injection therapy for frozen shoulder, please use the box below. You may enter up to 1000 characters. If you have nothing further to add, please leave blank and please press 'next' to finish.
